# The German national guideline “prevention of dysregulated screen media use in childhood and adolescence”

**DOI:** 10.1093/eurpub/ckaf125

**Published:** 2025-10-16

**Authors:** David D Martin, Silke A Schwarz

**Affiliations:** Chair of Medical Theory, integrative and anthroposophic Medicine, Institute of Integrative Medicine, University of Witten/Herdecke, Witten, Germany; Pediatric Oncology Department, Tübingen University Children’s Hospital, Tübingen University, Tübingen, Germany; Chair of Medical Theory, integrative and anthroposophic Medicine, Institute of Integrative Medicine, University of Witten/Herdecke, Witten, Germany

## Abstract

The increasing prevalence of digital media has raised concerns regarding its potential impact on child and adolescent development. In response, Germany has developed a National AWMF (Association of the Scientific Medical Societies) Guideline titled *"Prevention of Dysregulated Screen Media Use in Childhood and Adolescence"*. This guideline was created through a structured, interdisciplinary consensus process involving experts in pediatrics, psychiatry, psychology, and public health. Dysregulated screen media use is defined as problematic usage patterns in terms of duration, content, or function. The guideline introduces age-specific recommendations aligned with the 3-6-9-12 rule, discouraging any screen exposure before age 3, and progressively increasing screen time limits while maintaining strong parental supervision. For example, children aged 3–6 years should have no more than 30 minutes of screen time under parental guidance, while 9–12-year-olds are limited to 45–60 minutes, without daily use or personal game consoles. Overarching principles include limiting total screen time, avoiding screen use during meals, and discouraging its use as a behavioral tool. Emphasis is placed on parental involvement and awareness of school-related screen exposure. The guideline provides a comprehensive, developmentally informed framework aimed at promoting healthier digital habits in children and adolescents, while highlighting the ongoing need for research in a rapidly evolving digital landscape.

## Background and rationale

The rapid proliferation of digital technologies has led to increased screen media use among children and adolescents, raising concerns about potential negative impacts on their development and well-being. In Germany, the development of a National AWMF (Association of the Scientific Medical Societies in Germany) Guideline for “Prevention of Dysregulated Screen Media Use in Childhood and Adolescence” was initiated for several reasons, not least because the German Association of Pediatricians recommending no screens under the age of 3 years whereas WHO recommended no screens below the age of 2 [1–3]. The National AWMF Guideline also ended up not recommending screens under the age of 3 years.

## Methodology

Dysregulated screen media use is defined as problematic usage patterns in terms of time, content, or function. The guideline was developed using a structured consensus process involving experts from various relevant fields, including pediatrics, child and adolescent psychiatry, psychology, and public health. The German guideline is age-specific with the 3-6-9-12 rule:

The guideline provides age-specific recommendations for screen media use:

1. Children under 3 years: No screen media exposure is recommended
2. Children 3-6 years: If parents choose to introduce screen media, usage should be limited to a maximum of 30 min on individual days, always under parental supervision
3. Children 6–9 years: Recreational screen time should be limited to 30–45 min on individual days
4. Children 9–12 years: Recreational time limited to 45–60 minutes per day, no own game console, and not every day.

These recommendations are based on developmental considerations and the potential risks (such as myopia, mental health, sleep disruption, and developmental issues) associated with excessive screen time in early childhood.

## Overarching principles

The guideline emphasizes several overarching principles:

1. Less is better: In general, less screen time is considered more beneficial for children and adolescents
2. Parental involvement: Parents are encouraged to take an active interest in their children’s digital activities and provide critical guidance
3. No screens during meals: The guideline recommends avoiding screen use during meals, especially family meals, to promote social interaction and healthy eating habits.
4. Avoiding reward/punishment use: Screen time should not be used as a reward, punishment, or calming tool.
5. Keeping school screen time in mind: Total Screen time at school needs careful consideration.

## Conclusion

The German National AWMF guideline represents a significant effort to address the complex issues surrounding children’s digital media consumption. By providing evidence-based recommendations and emphasizing the importance of parental involvement and age-appropriate limits, the guideline aims to promote healthy development in the digital age. It further underscores the need for continued research and vigilance as technology continues to evolve and to shape the lives of children and adolescents.

## Data Availability

The data underlying this article are available at https://register.awmf.org/assets/guidelines/027-075l_S2k_Praevention-dysregulierten-Bildschirmmediengebrauchs-Kinder-Jugendliche_2023-09.pdf.
